# Barrel cortex VIP/ChAT interneurons suppress sensory responses in vivo

**DOI:** 10.1371/journal.pbio.3000613

**Published:** 2020-02-06

**Authors:** Amir Dudai, Nadav Yayon, Vitaly Lerner, Gen-ichi Tasaka, Yair Deitcher, Karin Gorfine, Naomi Niederhoffer, Adi Mizrahi, Hermona Soreq, Michael London

**Affiliations:** 1 The Edmond and Lily Safra Center for Brain Sciences (ELSC) and The Department of Neurobiology, The Life Sciences Institute, The Hebrew University of Jerusalem, Jerusalem, Israel; 2 The Edmond and Lily Safra Center for Brain Sciences (ELSC) and The Department of Biological Chemistry, The Life Sciences Institute, The Hebrew University of Jerusalem, Jerusalem, Israel; Ecole Polytechnique Federale de Lausanne, SWITZERLAND

## Abstract

Cortical interneurons expressing vasoactive intestinal polypeptide (VIP) and choline acetyltransferase (ChAT) are sparsely distributed throughout the neocortex, constituting only 0.5% of its neuronal population. The co-expression of VIP and ChAT suggests that these VIP/ChAT interneurons (VChIs) can release both γ-aminobutyric acid (GABA) and acetylcholine (ACh). In vitro physiological studies quantified the response properties and local connectivity patterns of the VChIs; however, the function of VChIs has not been explored in vivo. To study the role of VChIs in cortical network dynamics and their long-range connectivity pattern, we used in vivo electrophysiology and rabies virus tracing in the barrel cortex of mice. We found that VChIs have a low spontaneous spiking rate (approximately 1 spike/s) in the barrel cortex of anesthetized mice; nevertheless, they responded with higher fidelity to whisker stimulation than the neighboring layer 2/3 pyramidal neurons (Pyrs). Analysis of long-range inputs to VChIs with monosynaptic rabies virus tracing revealed that direct thalamic projections are a significant input source to these cells. Optogenetic activation of VChIs in the barrel cortex of awake mice suppresses the sensory responses of excitatory neurons in intermediate amplitudes of whisker deflections while increasing the evoked spike latency. The effect of VChI activation on the response was similar for both high-whisking (HW) and low-whisking (LW) conditions. Our findings demonstrate that, despite their sparsity, VChIs can effectively modulate sensory processing in the cortical microcircuit.

## Abstract 2

This study shows that despite their sparsity (about 0.5% of neurons), a population of interneurons in the mouse barrel cortex that express genes associated with both cholinergic and GABAergic signalling can effectively modulate sensory processing in the cortical microcircuit.

## Introduction

Cortical microcircuit research has been revolutionized by the development of advanced tools that enable the specific labeling and manipulation of genetically identified subpopulations of neurons [1,2]. One such subpopulation is the group of neocortical interneurons that express choline acetyltransferase (ChAT). Initial histological studies [3–5] identified these neurons as a very sparse group, constituting 0.5% of the cortical neuronal population. They are located mainly in layer 2/3 and have a predominantly bipolar structure. These cortical interneurons are a subgroup of a larger population of interneurons expressing vasoactive intestinal polypeptide (VIP). As such, the cortical VIP/ChAT interneurons (VChIs) can corelease both γ-aminobutyric acid (GABA) and acetylcholine (ACh) [5–11].

ACh modulates neuronal biophysical properties such as synaptic release probability and gain of potassium ion channels [12–20]. ACh also affects high-level cognitive functions such as attention and memory [21–27]. While cholinergic effects on the cortex have mainly been attributed to the cortical projections from the basal forebrain (BF) [28–31], there is evidence that up to 30% of the ACh in the cortex is local [28]. Thus, VChI release of ACh suggests that these neurons, despite their sparseness, may contribute to cortical information processing.

Since VChIs are a subgroup of the VIP^+^ population, it is important to examine their function in that context as well. Notably, changes in brain states significantly affect the VIP^+^ population activity, modulating the processing of sensory information [32–38]. VIP^+^ neurons directly inhibit the activity of the somatostatin-expressing (SST^+^) inhibitory interneurons, and therefore their activation mainly results in the disinhibition of cortical excitatory neurons [32,39–45]. However, although the VIP^+^ population has been extensively studied, little is known about the functional role of the VChI subgroup in vivo, and in particular about its involvement in sensory processing.

von Engelhardt and colleagues (2007) provided the main source of information about the physiology of VChIs when they genetically labeled the VChI subpopulation and recorded from it in vitro [5]. This study showed a low connection probability from cortical pyramidal neurons (Pyrs) to VChIs and no direct synaptic connections from VChIs to Pyrs and fast-spiking neurons. Later, Arroyo and colleagues (2012) demonstrated that optogenetic activation of the BF excites VChIs, which in turn causes inhibitory currents in Pyrs [46]. Recent studies combining optogenetics and electrophysiology in the mouse frontal and visual cortex showed that VChIs have a specific output connectivity pattern, as they preferentially target layer 1 and layer 6 neurons, as well as the SST^+^ subpopulation [9,10]. These studies also measured both nicotinic and GABAergic currents, confirming that VChIs do form synapses that release both ACh and GABA.

Here, we used ChAT-Cre mice and Cre-dependent viral vectors to label, tag, and manipulate VChIs in the barrel cortex. We performed electrophysiological recordings in anaesthetized and awake mice while applying sensory and optogenetic stimulations, and we used monosynaptic rabies tracing to find the VChI input connectivity pattern. We describe the response properties of the VChI population, as well as their input connectivity and their output effects in vivo. Our results demonstrate that VChIs are functionally integrated into the local and global circuit and that, despite their sparseness, they play an active and significant role in sensory processing.

## Results

### Cortical VChIs: Density and proportion

**Fig 1:**
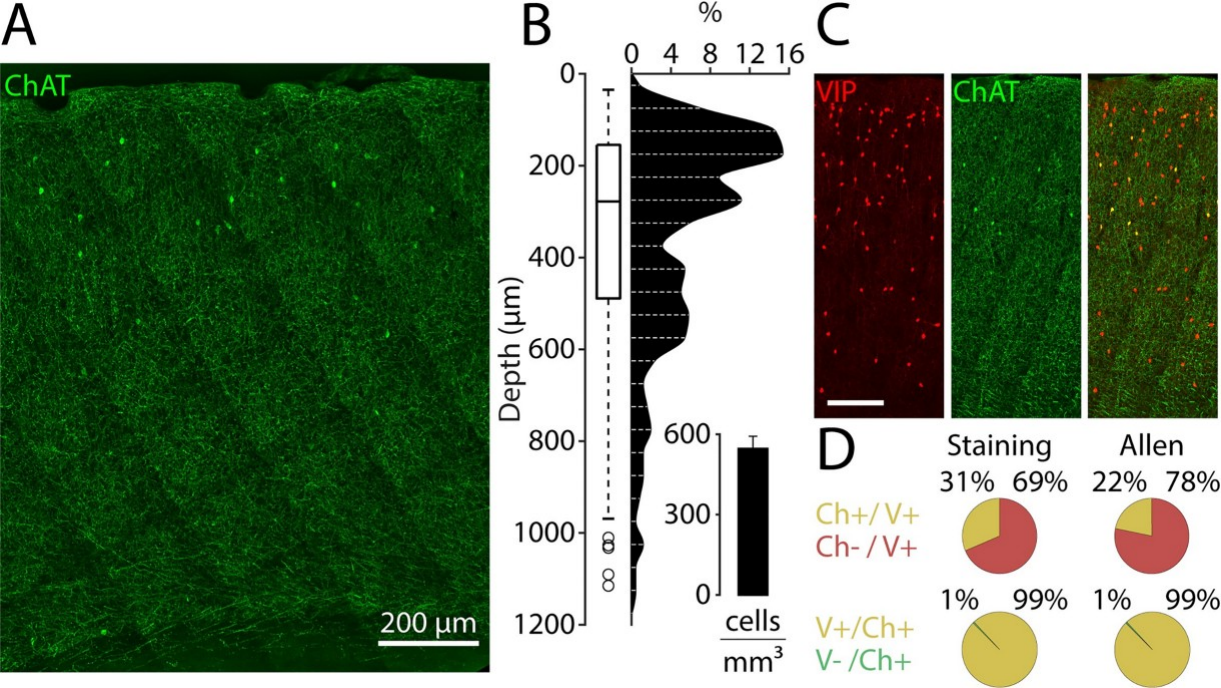
VChI cortical density and VIP/ChAT colocalization. (A) Cortex stained with ChAT primary antibody in a 30-μm–thick coronal slice (maximum intensity z-projection; Alexa 647). (B) VChI distribution across cortical depth (*n =* 261 cells; 3 mice). Inset: cell counts per cubic millimeter. (C) Cortex of a VIP-tdTomato mouse stained with ChAT (maximum intensity z-projection; Alexa 647). Left: tdTomato; Middle: ChAT; Right: overlay (scale: 100 μm). (D) Respective ratios of cortical ChAT^+^ and VIP^+^ neuronal populations. Left: staining data. Right: Allen Institute single-cell RNA-seq data (“Ch” = ChAT; “V” = VIP). ChAT, choline acetyltransferase; RNA-seq, RNA sequencing; VChI, VIP/ChAT interneuron; VIP, vasoactive intestinal polypeptide.

The estimates regarding the sparseness of VChIs vary between studies [7,9,11,47]. In order to obtain an independent estimate of the number and the spatial distribution of VChIs, we used immunostaining in wild-type (WT) mice (Fig 1A and 1B; see Materials and methods). The VChI population had higher density in layer 2/3 (first quartile 155 μm, median 278 μm, third quartile 489 μm; *n =* 261; 3 mice). An estimation of the number of VChIs across all layers yielded 546 ± 81 cells/mm^3^ (mean ± SD) in the barrel cortex. This is equivalent to 0.5% of the cortical neuronal population [48]. We quantified the proportion of ChAT^+^ cells expressing VIP and the proportion of VIP^+^ cells expressing ChAT (Fig 1C and 1D). The VIP^+^/ChAT^+^ colocalization was 99.4% ± 0.7%, meaning that all ChAT^+^ cells also expressed VIP. However, the relative proportion of ChAT^+^/VIP^+^ was 31.4% ± 6.0% (mean ± SD; 5 mice). Analysis of single-cell RNA sequencing (RNA-seq) data from the primary visual cortex and the anterior lateral motor cortex—provided by the Allen Institute [49]—yielded 99.3% (691/696 neurons) for the VIP^+^/ChAT^+^ colocalization and 21.8% (691/3,156 neurons) for the ChAT^+^/VIP^+^ colocalization.

### Targeting VChIs through the ChAT-Cre mouse model

**Fig 2:**
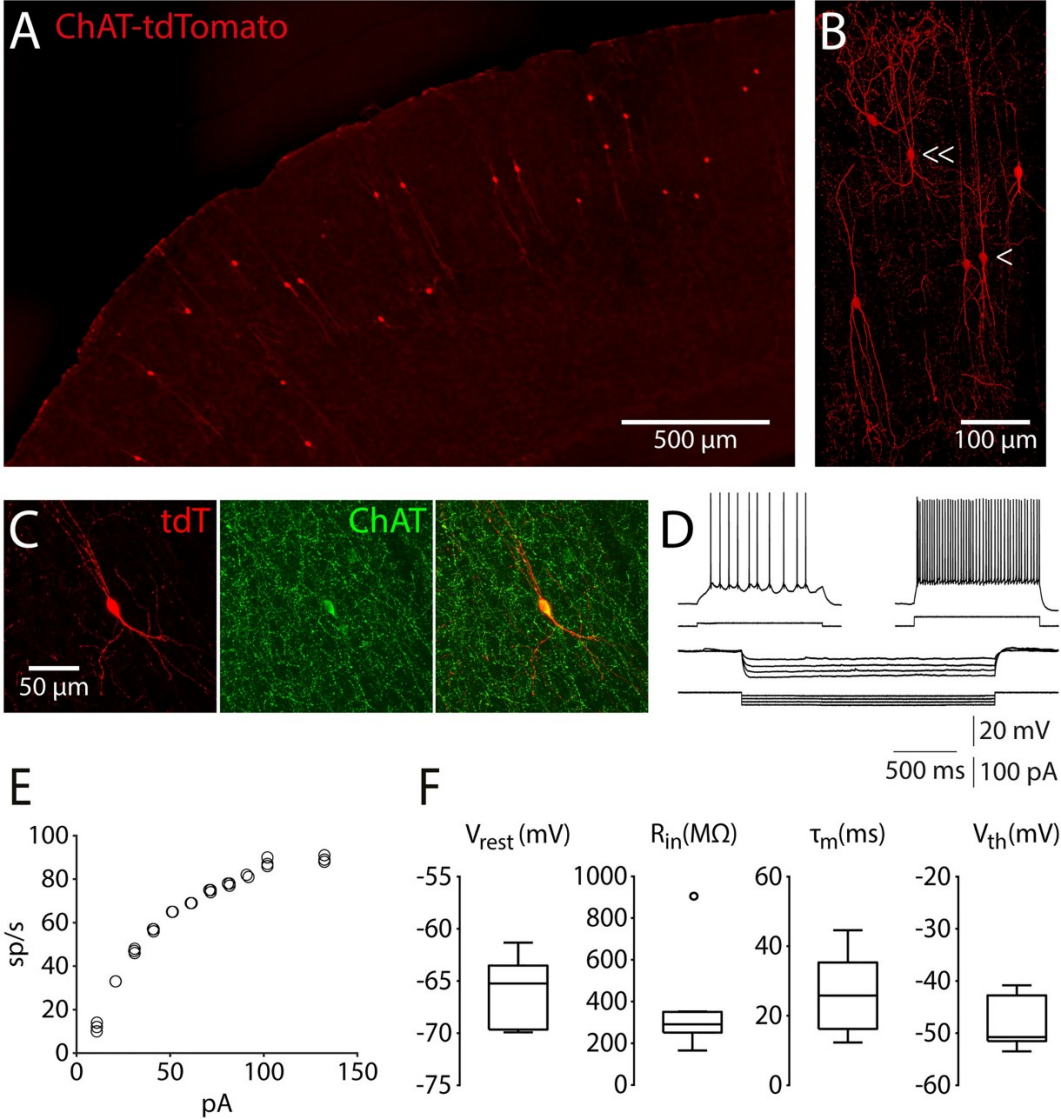
Anatomical and physiological properties of tdTomato-expressing cells in ChAT-tdTomato mice. (A) Image of a ChAT-tdTomato mouse cortex (30-μm coronal slice). (B) Three-dimensional rendering of VChIs in a coronal slice from a ChAT-tdTomato mouse (cube 300 × 700 × 150 μm; see S1 Video). (C) Expression of ChAT in cortical tdTomato-expressing cells. Left: tdTomato; Middle: ChAT staining (Alexa 648); Right: overlay. (D) Membrane potential responses to hyperpolarizing and depolarizing current steps of a cortical tdTomato-expressing cell. (E) F-I curve of the recorded cell shown in panel D. (F) Physiological properties of the recorded cells. τ_m_, membrane time constant; ChAT, choline acetyltransferase; F-I, frequency-current; R_in_, input resistance; sp/s, spikes per second; V_rest_, resting potential; V_th_, threshold potential; VChI, VIP/ChAT interneuron; VIP, vasoactive intestinal polypeptide.

We gained access to cortical VChIs by using ChAT-Cre mice (see Materials and methods). To validate the specificity of VChIs, we crossed ChAT-Cre mice with reporter mice conditionally expressing tdTomato [50] (Fig 2A–2C). In the crossed mice, the specificity of cortical tdTomato cells for VChIs was 98% ± 2% (ChAT^+^/tdTomato; 5 mice); however, the efficiency was 37% ± 3% (tdTomato/ChAT^+^; 5 mice; in agreement with previous reports [51]). This means that all tdTomato-expressing cortical cells are also ChAT^+^ and that about a third of the ChAT^+^ cells are marked with tdTomato. Fig 2B shows a three-dimensional rendering of VChIs in a coronal slice from a ChAT-tdTomato mouse (imaged with a confocal microscope from both sides of the slice and corrected for depth illumination gradients [52]; see S1 Video). Compatible with previous reports [5,53,54], we observed two morphologically distinct cell types of cortical VChIs in the ChAT-tdTomato mice: bipolar and multipolar cells. Whole-cell patch-clamp recordings in vitro were used to obtain the physiological properties and input–output relationship (frequency-current curve) of tdTomato-expressing cells (Fig 2D and 2E). The resting membrane potential of the tdTomato cells was −66 ± 1 mV, their input resistance (R_in_) was 340 ± 60 MΩ, the membrane time constant (τ_m_) was 26 ± 4 ms, and their threshold potential (V_th_) was −48 ± 1 mV (*n =* 10 cells; Fig 2F). We conclude that the ChAT-Cre mice provide specific access to VChIs in the cortex, since the anatomical and electrophysiological properties of VChIs in these mice are consistent with the properties found in previous works [5,51].

### Spontaneous firing rate and reliability of sensory-evoked responses of VChIs

**Fig 3:**
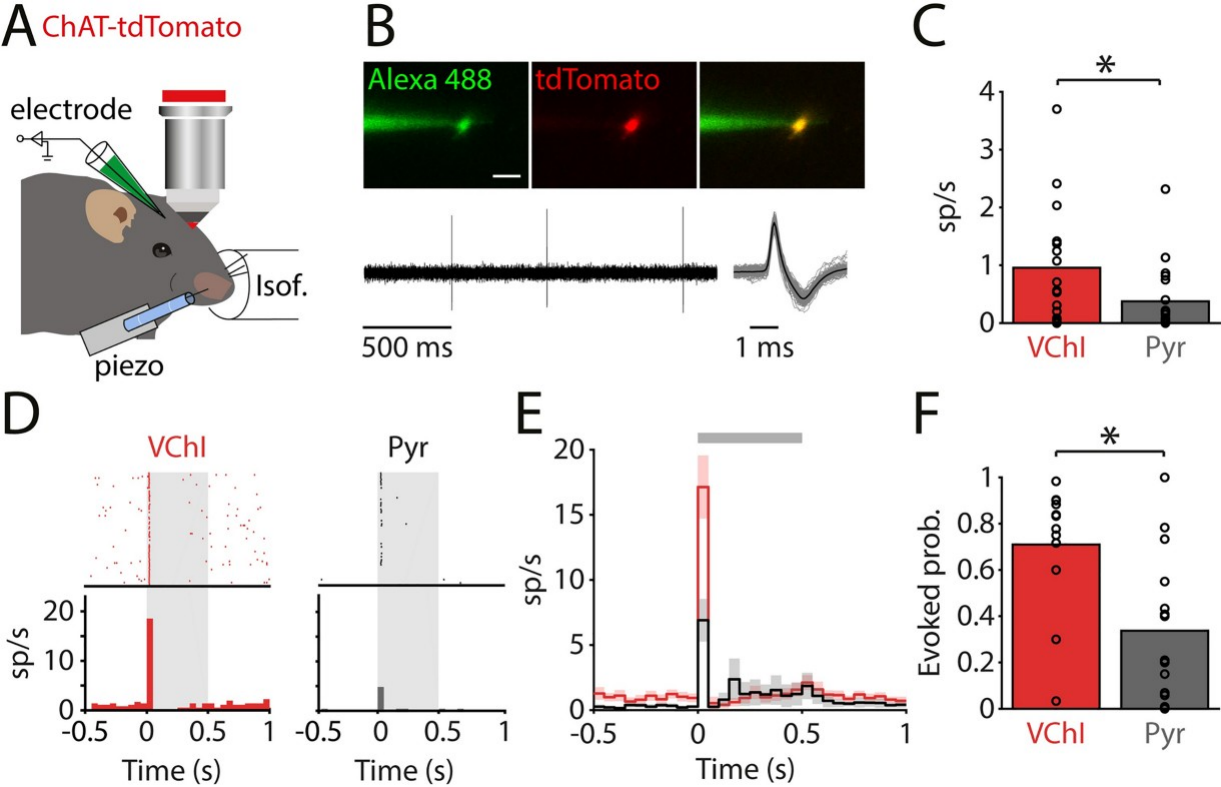
VChI spontaneous firing rate and reliability in response to sensory stimulation. (A) Illustration of the experimental system: two-photon guided cell-attached recordings from VChIs in an anaesthetized ChAT-tdTomato mouse with or without whisker deflection. (B) Top left (green): following electroporation, the recorded cell is filled with Alexa 488. Top middle (red): tdTomato expression of the recorded cell. Right: overlay (mean-intensity z-stack projection; scale: 20 μm). Bottom: spontaneous cell-attached recording from a VChI. (C) Spontaneous firing rate of VChIs and Pyrs. (D) Raster plot and PSTH of a VChI and Pyr during single-whisker stimulation (grey shaded area: piezoelectric whisker stimulation). (E) Population average PSTH of recorded VChIs and Pyrs (grey horizontal bar: piezoelectric whisker stimulation). (F) Probability of evoking a spike following the onset of the whisker stimulation. ChAT, choline acetyltransferase; Isof, isoflurane; PSTH, peristimulus time histogram; Pyr, pyramidal neuron; sp/s, spikes per second; VChI, VIP/ChAT interneuron; VIP, vasoactive intestinal polypeptide.

We studied the spontaneous firing rate of VChIs using two-photon targeted cell-attached recordings [55,56] (see Materials and methods) from tdTomato-labeled cells in lightly isoflurane-anesthetized mice (Fig 3A and 3B). Cells were electroporated at the end of the experiment for targeting validation (only cells filled with Alexa 488 were included in analysis). The spontaneous activity of VChIs was 1.0 ± 0.2 spikes/s. In comparison, the spontaneous firing rate of putative Pyrs was significantly lower, 0.4 ± 0.1 spikes/s (*n*_VChI_ = 17, *n*_Pyr_ = 20, unpaired *t* test, *t*[35] = 2.2, *p =* 0.03; Fig 3C), consistent with previous reports of sparse activity of Pyrs in layer 2/3 in the barrel cortex [57,58]. To quantify the response of VChIs to sensory stimulation, we recorded their activity in a loose-patch configuration while delivering a square pulse deflection (0.5 s) to the principal whisker (PW) using a piezoelectric actuator. Raster plot examples and the corresponding peristimulus time histograms (PSTHs) are shown in Fig 3D. In response to sensory stimulation, VChIs sharply increased their firing rate. Analysis of the population showed that VChIs are more sensitive to sensory stimulation than layer 2/3 Pyrs (Fig 3E; 50 ms bin; VChI: 17 ± 2 spikes/s; Pyr: 7 ± 2; *n*_VChI_ = 12, *n*_Pyr_ = 15, unpaired *t* test, *t*[25] = 3.6, *p =* 0.001). The temporal delay observed in the VChIs’ response (25 ± 2 ms) suggests that VChIs receive excitatory input from local excitatory neurons or directly from thalamic projections. The probability of generating action potential in the 50 ms following stimulation onset was 0.7 ± 0.1 for VChIs, compared to 0.3 ± 0.1 for Pyrs (*n*_VChI_ = 12, *n*_Pyr_ = 15, unpaired *t* test, *t*[25] = 3.2, *p =* 0.004; Fig 3F). To conclude, we found that the spontaneous rate of VChIs is low, yet still higher than that of the Pyrs in their vicinity. In addition, we found that VChIs are well integrated into the network and receive strong input from the sensory pathway. This input evokes a highly reliable response of VChIs, which may participate in processing the afferent tactile information.

### Long-range input connectivity pattern of VChIs

**Fig 4:**
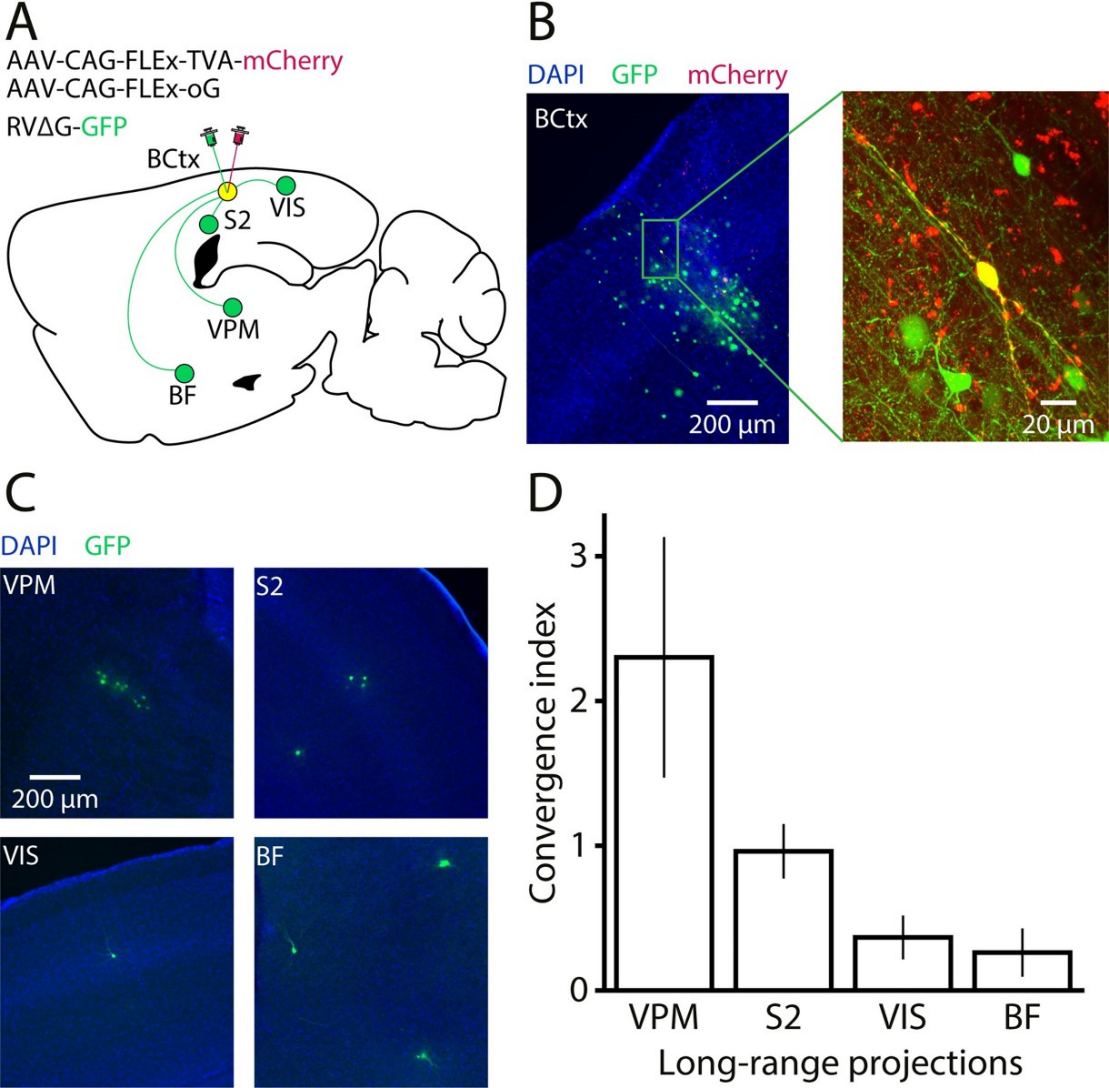
Rabies *trans*-synaptic tracing from VChIs in the barrel cortex. (A) Illustration of the experimental procedure and the distal areas innervating the VChIs in the BCtx. The identified presynaptic distal areas of the VChIs are the VPM, S2, VIS, and the BF. (B) An example image of a VChI starter cell (expressing both mCherry and GFP) located in layer 2/3. Inset: zoom-in image showing the bipolar morphology of the cell. (C) Examples of input cells (expressing GFP) from the 4 identified distal areas. (D) The CI distribution quantifying the relative number of input cells per starter cell in all identified input areas. BCtx, barrel cortex; BF, basal forebrain; ChAT, choline acetyltransferase; CI, convergence index; GFP, green fluorescent protein; RVΔG, G-deleted rabies virus; S2, secondary somatosensory cortex; VChI, VIP/ChAT interneuron; VIP, vasoactive intestinal polypeptide; VIS, primary and secondary visual areas; VPM, ventral posteromedial thalamic nucleus.

To reveal the anatomical presynaptic inputs to VChIs, we used monosynaptic *trans*-synaptic rabies tracing [59]. We injected a mix of a Cre-dependent optimized glycoprotein (oG) required for the rabies virus envelope [60,61] and a Cre-dependent avian tumor virus receptor A (TVA) [62] into the barrel cortex (Fig 4A). Two weeks later, we injected an EnvA-Pseudotyped G-deleted rabies virus into the exact same site. We euthanized the animals for histology 5 d later and mapped the number of green fluorescent protein (GFP)-expressing neurons (the presynaptic inputs of the VChI seeds) and their locations. To avoid false-positive input cells in the vicinity of the injection point [62], we only considered long-range connections from cells outside the primary somatosensory cortex, even though we estimate that a significant portion of the local GFP^+^ cells are indeed presynaptic. Quantifying the exact number of cells within the barrel cortex that were labeled both with GFP (rabies) and mCherry (TVA) yielded 4.5 ± 1.4 starter cells per animal, all of which were restricted to the barrel cortex. In total, we obtained 27 starter cells from 6 mice (Fig 4B). Overall, 108 input cells were detected in 4 distal areas, and for each area we calculated the convergence index (CI; defined as the number of input neurons in a brain region per starter cell; Fig 4C and 4D). The areas in which GFP^+^ cells were found are the ventral posteromedial thalamic nucleus (VPM; CI 2.3 ± 0.9), the secondary somatosensory cortex (S2; 1.0 ± 0.2), the primary and secondary visual areas (0.4 ± 0.2), and the BF structure consisting of inputs from the nucleus basalis and the substantia innominata (0.3 ± 0.2). To conclude, the long-range projections innervating the VChIs mainly originate in the VPM and S2, but considerable input also arrived from the visual cortex and the BF. Along with the projections from the local circuitry, the direct input from the VPM likely contributed to the latency and reliability of the whisker-evoked responses reported in Fig 3.

### Optogenetic activation of VChIs inhibits the sensory response of excitatory neurons

**Fig 5:**
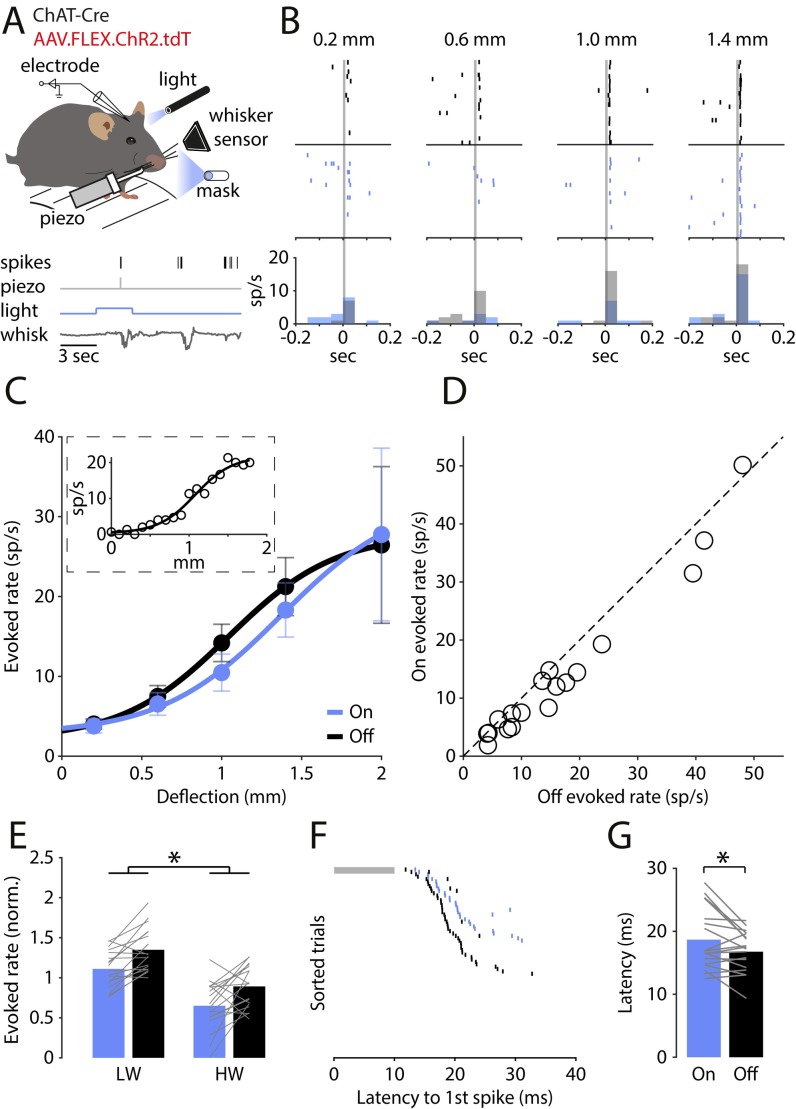
VChIs inhibit the sensory response elicited by whisker deflection. (A) Illustration of the experimental system: cell-attached recordings are performed from putative excitatory cells in an awake, head-fixed mouse. In each trial, a piezoelectric deflection in a random amplitude (0.2, 0.6, 1.0, or 1.4 mm) is delivered to the whiskers, and optogenetic stimulation is either delivered (“On” trial) or not (“Off” trial). (B) Example of the response of a single cell to the 4 whisker deflection amplitudes in the Off and in the On trials. (C) Population average of the response of all cells to the whisker deflections in the Off and On trials. Inset: example of the sigmoidal response of a cell in which the whiskers were deflected in 19 different amplitudes. (D) The average evoked rate for each cell in the intermediate range of the sigmoidal curve. (E) Evoked rate in the Off and On trials in LW and HW trials. (F) The response of a cell to whisker deflection, sorted by the lag to the first deflection-evoked spike (grey horizontal bar: piezoelectric stimulation). (G) The latency to the first evoked spike in the On and in the Off trials across the population. ChAT, choline acetyltransferase; HW, high whisking; LW, low whisking; sp/s, spikes per second; VChI, VIP/ChAT interneuron; VIP, vasoactive intestinal polypeptide.

To test the functional role of VChIs in the processing of sensory information, we combined loose-patch recordings from putative excitatory cells in awake head-fixed mice with optogenetics. We injected ChAT-Cre mice with Cre-dependent channelrhodopsin2 (ChR2) for a specific expression of ChR2 in VChIs (S1A Fig; see Materials and methods). We first validated the selectivity and efficiency of the expression of ChR2 with immunostaining as well as with electrophysiological recordings in vitro and in vivo (S1 Fig). This specific expression of ChR2 in VChIs allowed us to detect their inhibitory role in the local circuitry of the barrel cortex (Fig 5 and S2 Fig). We optogenetically activated the VChIs while stimulating the whiskers in various deflection amplitudes in awake, head-fixed mice (Fig 5A). A whisker deflection was delivered for 10 ms with an amplitude that was randomly selected in each trial (0.2, 0.6, 1.0, and 1.4 mm). The trials were also randomly selected to include a 3-s optogenetic activation of VChIs that started 2 s before the sensory stimulus (“On”) or not (“Off”; Fig 5A). In all trials, a “masking light” of 3 s was delivered in the background, controlling for visual perception of the light stimulation (S2H and S2I Fig; see Materials and methods). Fig 5B shows responses (raster plots and PSTHs) of a putative excitatory cell to each whisker deflection amplitude, in the On and Off conditions. VChI activation led to a detectable decrease in the evoked response for deflection amplitudes of 0.6 and 1.0 mm.

In order to evaluate this effect for the population, we first systematically quantified the evoked response of neurons by deflecting the whiskers over a range of 19 amplitudes. The response curve was well described by a sigmoidal function (see Fig 5C inset). We therefore fit a sigmoidal curve to the whisker stimulation responses for both the Off and On conditions obtained from a population of 18 neurons and, in addition, a separate population of 9 neurons that received only a strong whisker deflection of 2.0 mm (Fig 5C). A comparison of the sigmoidal curves for both conditions shows that the sensory response curve in the On condition is shifted to the right (toward higher deflection amplitudes) compared to the Off condition. This shift indicates inhibited responses of excitatory cells to whisker stimulation amplitudes in the range of 0.6–1.4 mm, which is less effective for larger deflection amplitudes. This may provide a mechanism that adjusts the operational range of neurons without affecting their gain. The scatter plot in Fig 5D compares the average PSTH peak amplitude for the intermediate whisker deflections (0.6, 1.0, and 1.4 mm) between the light Off and On conditions. For this range of whisker deflections, there is a significant reduction in the peak PSTH response (Off: 17 ± 3 spikes/s, On: 14 ± 3 spikes/s; *n =* 18, paired *t* test, *t*[17] = 4.3, *p =* 0.0005). It has been reported that spontaneous whisking can cause ACh release in the cortex from BF cholinergic projections as well as activate local VIP^+^ interneurons [16,38,39]. Thus, the concentration of ACh in the cortex is expected to increase during spontaneous whisking, and the activity of VIP^+^ neurons will also be elevated. If the effect of VChIs is achieved via their ACh release, then it is possible that, during high-whisking (HW) activity, their effect will be masked. Similarly, as part of the VIP^+^ population, if the activity of VChIs increases during HW, their relative contribution will be less significant while activating them with ChR2. Thus, it is important to check whether the sensory inhibition caused by VChIs is modulated by spontaneous whisking. We classified trials into low whisking (LW) and HW, as measured by the whisker sensor positioned on the nonstimulated whisker pad. We pooled together all intermediate whisker deflections for each group and normalized the evoked rate in each condition by the mean evoked response of the cell across conditions (Fig 5E). We found a significant main effect of whisking and of optogenetics with no effect of interaction (On_LW_: 1.11 ± 0.05, Off_LW_: 1.35 ± 0.06, On_HW_: 0.65 ± 0.07, Off_HW_: 0.89 ± 0.06; two-way repeated-measures ANOVA; a main effect of optogenetics F[1,17] = 20.82, *p =* 0.0003, η^2^_G_ = 0.17; a main effect of whisking F[1,17] = 18.36, *p =* 0.0005, η^2^_G_ = 0.43; no optogenetics × whisking effect of interaction F[1,17] = 0.002, *p =* 0.96, η^2^_G_ = 1.5 × 10^−5^). Finally, we measured the latency to the first spike following whisker stimulation. Fig 5F shows the response of a neuron in the 40 ms following whisker stimulation (trials are sorted by the latency to the first evoked spike). We found that the average latency in the Off trials was shorter than the average latency in the On trials, in which the VChIs were optogenetically stimulated (Latency_On_ 18.7 ± 1.1 ms, Latency_Off_: 16.9 ± 0.8 ms; paired *t* test, *t*[17] = 2.44, *p =* 0.03). This longer average latency provides additional evidence for the inhibitory effect of VChIs on the sensory response.

## Discussion

The physiology of cortical VChIs has not been explored in vivo, and thus very little is known about their activity and role in network dynamics. We identified individual VChIs in the barrel cortex, and recorded their spontaneous and stimulus-evoked activity in a loose-patch configuration. Our findings revealed that VChIs have a higher firing rate and respond with higher fidelity to whisker stimulation than Pyrs. We then examined the long-range input connectivity to the VChIs and observed a strong direct thalamic input, as well as inputs from S2, visual areas, and the BF. Optogenetic activation of VChIs combined with sensory stimulation in awake, head-fixed mice revealed that VChIs play a role in inhibiting the response to whisker stimulation both in LW and HW states, decreasing the spiking response of excitatory neurons and increasing the latency of the whisker-evoked spike.

### Using ChAT-Cre mice to study VChIs

Even though VChIs constitute only 0.5% of all cortical neurons, crossing ChAT-Cre with Cre-dependent reporter allows for specific targeting of VChIs in the cortex. Furthermore, the morphology observed and the measured biophysical properties of VChIs indicated that ChAT-Cre mice are highly suitable for exploring these cells. The existence of 2 types of dendritic tree geometries (bipolar and multipolar) may indicate that the VChI population can be further divided into subtypes of neurons. Further investigation using single-cell sequencing technologies [63–65] combined with large-volume structural analysis [52] may help resolve this question. Ultimately, any findings provided by these methods should be supported by physiological and functional evidence.

### Input–output properties of VChIs

Our rabies tracing data indicate that the primary long-range source of synaptic inputs to VChIs is the VPM, supporting the VChI response reliability in our physiological results. In addition, anatomical and physiological studies suggest that VChIs receive synaptic input from BF projections [46,66]. Our tracing data provide new evidence for this cholinergic–cholinergic connection as well as for inputs from cortical visual areas and S2. Current viral-based rabies techniques are limited in their ability to maintain both high efficiency and a low false-positive signal when considering both local and long-range connections. We and others have shown that, due to the high sensitivity of the TVA, rabies tracing will inevitably result with several dozen false-positive neurons near the injection site [62,67]. Here, in order to avoid any false-positive signals, we limited our quantitative analysis to long-range inputs. Qualitatively, however, we can estimate that the vast majority of the presynaptic input neurons to VChIs arise locally (within a 300-μm radius).

Our in vivo data show that VChIs shift the sensory response curve of excitatory neurons toward higher stimulation amplitudes. Our results correspond with Arroyo and colleagues (2012), who show in vitro that the activation of VChIs causes a barrage of inhibitory postsynaptic currents (IPSCs) in neighboring Pyrs in the sensorimotor cortex [46]. Two recent studies [9,10] used optogenetics and electrophysiology in vitro to study the local output connectivity pattern of cortical VChIs. Interestingly, both studies demonstrated a release of ACh from VChI output synapses. Obermayer and colleagues (2019) recorded from cells in the frontal cortex and reported direct nicotinic excitatory synapses from VChIs to layer 1 and layer 6 neurons, and that approximately 15% of the target cells also received GABAergic inhibitory input from VChIs. In addition, they found subgroups of local interneurons that received cholinergic excitatory input. Granger and colleagues (2018), who recorded from the frontal and visual cortex, showed that the elicited postsynaptic currents were predominantly GABAergic in neurons across all cortical layers. However, VChIs targeted SST^+^ interneurons with higher probability than any other neuronal subtype. New technologies monitoring neurotransmission [68] and neuromodulation release [69] may shed new light on the complex synaptic input–output connectivity map of VChIs in vitro and in vivo.

It is worth noting that a substantial inhibitory effect was also observed in another sparse population of cholinergic interneurons [70]. The striatal cholinergic interneurons (which do not express VIP) compose less than 1% of the striatal neurons. They show a very specific input–output connectivity pattern: they are not innervated by either medium spiny neurons (MSNs) or parvalbumin-expressing interneurons [71–73]; however, they form local connections with GABAergic interneurons such as neurogliaform [74]. Similar to VChIs, they also receive direct thalamic innervation [75], and despite their sparse density, optogenetic activation of the striatal cholinergic interneurons promotes substantial inhibitory effect to the MSNs [70].

The anatomical and physiological properties of the cortical VIP^+^ cells [39,43,44,76], as well as their long-range inputs, are similar to those of VChIs [77–80]. However, VChIs constitute approximately 30% of the VIP^+^ cells, and most studies of VIP^+^ population do not distinguish between VIP^+^/ChAT^+^ and VIP^+^/ChAT^−^. Future experiments, which are feasible with the advanced genetic tools nowadays, should probe and compare these two distinct subpopulations. Physiologically, many recent studies involving perturbation of VIP^+^ neurons demonstrated that activation of VIP^+^ neurons predominantly leads to disinhibitory effects on cortical activity, since they reduce tonic inhibition (mainly of SST^+^ cells) [32,39–42]. We, however, show that the net effect of VChI activation during sensory stimulation is predominantly inhibitory. The previous result and the evidence that VChIs indeed release ACh [9,10] lead us to hypothesize that the VChIs are a functionally distinct subpopulation.

## Materials and methods

### Ethics statement

All experiments were approved by the Institutional Animal Care and Use Committee (IACUC) of The Hebrew University of Jerusalem, which follows the National Research Council (US) Guide for Care and Use of Laboratory Animals (NS-18-15669-4).

### Mice

Throughout the paper, we used B6;129S6-Chat^tm2(Cre)Lowl^/J^+/+^ transgene (ChAT-Cre, stock number 018957, The Jackson Laboratory, Bar Harbor, ME). For the targeted-patch experiments (in vitro and in vivo), we cross-bred these mice with B6.Cg-Gt(ROSA)26Sor^tm14(CAG-tdTomato)Hze^/J^+/+^ transgene (Ai14 tdTomato, stock number 007914, The Jackson Laboratory). Adult mice (8–16 wk) from both sexes were used for all experimental procedures. For estimating the relative ratio of ChAT^+^ cells from VIP^+^ cells, we used Vip^tm1(cre)Zjh/J^ transgene (VIP-Cre, stock number 010908, The Jackson Laboratory) crossed with Ai14 tdTomato. This mouse line was shown to be highly specific and efficient in labeling cortical VIP^+^ cells [44,81].

### Immunostaining

We employed the following protocol of ChAT immunofluorescence for mouse brain sections (30 μm): first, permeabilization with PBS-TritonX-100 (1 h at room temperature, mild shaking); next, blocking in PBS-TritonX-100/5% Normal Donkey Serum (NDS; 1 h); then, incubation of the sections in primary antibody anti-ChAT 1:150 (Ab144p, MilliporeSigma, Burlington, MA) in PBS with Tween20/3% NDS (over 3 nights at 4°C, mild shaking); and last, incubation of the sections in the secondary antibody donkey anti-goat Alexa 647 1:500 (Jackson ImmunoResearch, West Grove, PA; 2 h at room temperature, mild shaking). We mounted the sections and imaged them with a FV-10i (Olympus, Tokyo, Japan) confocal microscope.

### Surgery and viral vector injections

All viral vectors were administered into the right barrel cortex (coordinates relative to Bregma: 1.5 mm posterior, 3.3 mm lateral, and 0.5 mm deep) using a Nanoject III apparatus (Drummond Scientific, Broomall, PA) under isoflurane anesthesia (2%–3%). For the optogenetics experiments, we injected 500 nl of AAV1-CAGGS-FLEX-rev-ChR2-tdTomato (1 × 10^13^ genomic copies per mL [82]) and installed a custom-made headpost using dental cement. Three weeks post injection, we made a 1-mm–diameter craniotomy (leaving the dura intact) near the injection site. The exposed brain was kept moisturized throughout the surgery (and later, the experiment) with the following extracellular solution (in mM): 150 NaCl, 2.5 KCl, 10 HEPES, 2 CaCl_2_, 1 MgCl_2_ (pH 7.3, adjusted with HCl/NaOH; 300 mOsm).

### Rabies tracing

We constructed the pAAV-CAG-FLEX-oG (as previously described [61]) using molecular cloning based on polymerase chain reaction (PCR) and restriction enzymes (New England Biolabs, Ipswich, MA). We amplified the oG with PCR from pAAV-EF1a-DIO-oG (Addgene Plasmid #74290; a gift from Edward Callaway [60]) and then subcloned it into pAAV-CAG-FLEX-RG (Addgene #48333; a gift from Liqun Luo [62]; digested with SalI and AscI). The TVA plasmid for the pAAV-CAG-FLEX-TVA-mCherry was a gift from Liqun Luo [62]. AAV vectors containing CAG-FLEX-TVA-mCherry (2 × 10^13^ genomic copies per mL) and CAG-FLEX-oG (1 × 10^12^ genomic copies per mL) were produced by the ELSC vector core facility. A mixture of a 0.2 ml AAV2-CAG-FLEX-TVA-mCherry and AAV2-CAG-FLEX-oG was stereotaxically injected into the same coordinates described previously (injected in a tilt of 20 degrees). The EnvA-Pseudotyped G-deleted rabies virus (2 × 10^11^ infectious particles per mL) was produced with the protocol described by Wickersham and colleagues [59] and Osakada and Callaway [83].

### In vitro electrophysiology

Slices were obtained at 35°C to 37°C, with the Campden 700-smz slicer and ceramic blades (Campden Instruments, Loughborough, UK). The details of the hot slicing method have been previously described [84]. The slicing, incubation, and bath solution was composed of (in mM) the following: 126 NaCl, 2.5 KCl, 1.25 NaH_2_PO_4_, 2 MgSO_4_, 10 glucose, 26 NaHCO_3_, and 2 CaCl_2_, bubbled with 95% O_2_/5% CO_2_.

Whole-cell patch-clamp recordings were done using an Olympus BX61WI (Olympus, Tokyo, Japan) microscope at room temperature. VChIs were identified using tdTomato fluorescence emitted while whole-field arc lamp illumination (U-LH100HG; Olympus) was applied and filtered (emission: 605–685 nm, excitation: 530–588 nm). For the optogenetics in vitro experiment, we used blue LED whole-field illumination (Prizmatix, Holon, Israel). Borosilicate glass microelectrodes (4–10 MΩ) were pulled in a Narishige PC-10 puller (Narishige, Tokyo, Japan) and filled with the following intracellular solution (in mM): 130 K-gluconate, 20 KCl, 10 Na_2_-phosphocreatine, 10 HEPES, 0.2 EGTA, 4 Mg-ATP, 0.3 Na_2_-GDP (pH 7.2, adjusted with HCl/NaOH; 298 mOsm). The electrodes were guided under DIC optics. Current-clamp recordings were done using a Multiclamp 700B amplifier (Molecular Devices, San Jose, CA), digitized at 20 kHz (NI PCI-6251; National Instruments, Austin, TX), and custom-written software based on LabVIEW (National Instruments).

### In vivo electrophysiology

Targeted-patch experiments were started with a detection of the PW. We recorded local field potential (LFP) signals 150–200 μm below the cortical surface with 0.5–2 MΩ borosilicate microelectrodes (Harvard Apparatus, Holliston, MA) pulled in a Narishige PC-10 puller (Narishige, Tokyo, Japan) and filled with the extracellular solution described earlier. The electrode was inserted into the brain with high pressure: we touched the dura with a positive pressure of 80–100 mBar and repeatedly pushed and pulled the pipette until the dura was penetrated, as indicated by the change in observed resistance. At that point, we decreased the pressure to 30–40 mBar. We placed a single whisker in a glass capillary tube attached to a piezoelectric actuator (E-650; Physik Instrumente [PI], Karlsruhe, Germany), deflected the whisker repeatedly (2.0-mm deflection from rostral to caudal for 500 ms) every 5 s, and averaged the LFP evoked responses. We switched the stimulated whisker until a quick (approximately 13 ms) change in the LFP voltage signal was observed. In most of the experiments, C2 and D2 were detected as the PWs. When we looked for cells, we used 4–10 MΩ electrodes, filled with a mix of extracellular solution (described earlier) and Alexa Fluor 488 dye (Thermo Fisher Scientific, Waltham, MA; 50 μM). Then, using a galvo-galvo two-photon imaging system (Sutter movable objective microscope, MOM; MScan software, Sutter Instrument, Novato, CA), we detected VChIs based on the tdTomato fluorescent signal. Images of the cells (460 × 240 pixels; 7.25 Hz) were acquired at 940 nm with a Ti:Sapphire laser (Vision II, Coherent, Santa Clara, CA) through a 16X, 0.8 NA water immersion objective (Nikon, Tokyo, Japan). Following that, we performed loose targeted-patch recordings of VChIs and stimulated the PW with the same stimulation protocol described earlier. At the end of each experiment, we filled the recorded cells with the fluorescent dye. We electroporated the cell membrane by slowly increasing the capacitance compensation. Once oscillations start, the capacitance compensation mechanism injects large current, which is immediately stopped by the oscillation prevention feedback mechanism (which happens around 8–10 pF compensation). Only filled cells with overlapping tdTomato and Alexa 488 were included in analysis (nonoverlapping cells were used as putative Pyrs). The experiments were performed under the lightest possible isoflurane anesthesia (0.5%–1%). The respiration cycle was monitored (OMEGA Engineering, Norwalk, CT), and only segments above 90 breaths/min on average were included in analysis.

Electrophysiological cell-attached recordings with optogenetics were done in head-fixed, awake mice. The mice were able to walk on a treadmill while we recorded from cells 200–400 μm below the cortical surface. Every 15 s, we stimulated a few whiskers for 10 ms with a piezoelectric actuator attached to a custom-designed plastic deflector. The deflection amplitude was randomized for each trial (0.2, 0.6, 1.0, and 1.4 mm from rostral to caudal). On a different set of cells, the same deflection protocol was used with a 2.0-mm deflection solely. Half of the trials were preselected randomly to apply optogenetic stimulation to VChIs (3-s continuous light pulse, as previously used for BF cholinergic activation [18,26]; 15 mW/mm^2^ using a 473-nm laser; Changchun New Industries Optoelectronics, Changchun, China) through a 16X, 0.8 NA water immersion objective (Nikon, Tokyo, Japan). The optogenetic stimulation started 2 s before the piezoelectric stimulation. Another light source (“masking light”; 470-nm LED; M4703L Thorlabs, Newton, NJ) was activated in all trials simultaneously with the optogenetic stimulation or at the same time respectively in the Off trials, controlling for visual perception of the optogenetic light. We recorded the walking of the animal using a reflective optical encoder (HEDR-5420-ES214, Avago Technologies, Yishun, Singapore) attached to the treadmill. The trials in which the animal walked more than 2 cm/s on average were excluded from the analysis. In addition, we traced mouse whisking throughout the experiment using an infrared reflective sensor (HOA1405-002; Honeywell, Charlotte, NC) that was placed on the contralateral side of the stimulated whisker pad.

All in vivo electrophysiological recordings were obtained using a Multiclamp 700B amplifier (Molecular Devices, San Jose, CA), digitized at 25 kHz (NI PCI-6321; National Instruments, Austin, TX). All data were acquired using Axograph X (Axograph Scientific, Sydney, Australia) and analyzed using MATLAB (MathWorks, Natick, MA).

### Data analysis and statistics

#### Allen Institute single-cell RNA-seq data

The data was downloaded from the Allen Brain “Cell Types” Atlas (https://celltypes.brain-map.org/rnaseq/mouse/v1-alm). We queried for the expression of ChAT and VIP (both in exons and introns) across all GABAergic cells in the data set. We defined a ChAT- or VIP-expressing cell as a cell in which more than 20 counts of mRNA were detected. Then, we checked the relative proportion of ChAT cells within the VIP^+^ population and the expression of VIP in all ChAT^+^ cells.

#### In vitro electrophysiology

All analyses were performed using MATLAB (MathWorks). In addition to the resting membrane voltage, we calculated the following electrophysiological parameters for each cell: the R_in_ was estimated by the slope of the current-voltage curve of the hyperpolarizing current steps; the τ_m_ was evaluated using 100 repetitions of 30 pA current steps and was estimated using the “peeling” method [85]; and V_th_ was defined as the voltage by which the change in voltage crossed 20 mV/ms.

#### Rabies tracing

We used ImageJ software [86] for counting cells in the images acquired (in each channel, we adjusted the brightness and contrast manually). We evaluated the number of inputs to VChIs in the barrel cortex from each brain region using the CI. This index was defined as the number of presynaptic GFP^+^ cells found in a brain region divided by the number of starter cells in this mouse. We used the Allen Brain Adult Mouse Reference Atlas (http://atlas.brain-map.org/) for region detection.

#### In vivo electrophysiology

All analyses were performed using MATLAB. In vivo cell-attached recordings were first high-pass filtered above 100 Hz. Then, by manually observing the spiking signal, we chose the threshold to be half the size of the average spike in the trace. The spike rate for each cell in a specific time bin was defined as the number of spikes in the time bin divided by the number of trials and the duration of the bin (50 ms, unless otherwise specified). The evoked probability was defined as the probability of having at least one spike in the first bin following whisker stimulation. The evoked rate as function of the deflection was modeled using the following sigmoidal fit: y = a + (b / [1 + exp (c * x + d)]). For the behavioral state analysis, we generated a distribution of the whisking values in the 2 s prior to whisker stimulation and assigned each trial to LW versus HW state in relation to the median whisking value for each mouse. We normalized the evoked response of a cell in each condition by dividing the responses by the mean evoked rate of the cell across conditions. We tested for normality with the Shapiro-Wilk test and for variance equality with Bartlett’s test. Then, we used two-way repeated-measures ANOVA to check for main effects and interaction between the optogenetics and the behavioral state conditions.

Unless otherwise reported, we used a two-tailed Student *t* test with *p <* 0.05 as the significance level. All data are reported as mean ± SEM unless otherwise specified. Paired- and unpaired-sample *t* tests were used when 2 groups were compared, based on the dependence between the analyzed groups.

## Supporting information

S1 Video *Related to Fig 2*. **Three-dimensional rendering of VChIs in a coronal slice from a ChAT-tdTomato mouse.** The slice was imaged with a confocal microscope from both sides of the slice and corrected for gradient illumination with Intensify3D [52] (300 × 700 × 150 μm cube; width × height × depth).
(MP4)

S1 Fig *Related to Fig 5*. **VChIs reliably express ChR2.** (A) Expression of a Cre-dependent ChR2-tdTomato virus in a ChAT-Cre mouse following injection to the barrel cortex (areas: cortex, striatum, and BF). (B) Immunostaining for ChAT (Alexa 647) in a ChAT-Cre mouse expressing ChR2-tdTomato. (C) In vitro responses to light stimulation of a ChR2-expressing neuron. An intensity of 1 mW/mm^2^ evoked a spiking response, whereas 0.1 mW/mm^2^ yielded only subthreshold depolarization. (D) In vivo targeted cell-attached recording from a VChI expressing ChR2. The raster plot (top) and PSTH (bottom; 5-ms bin) show sharp elevation of firing rate in response to light stimulation. Mean-intensity z-projection of the targeted cell (scale: 20 μm). (E) Three trials from panel D (blue area: light stimulation).
(TIF)

S2 Fig *Related to Fig 5*. **Two-photon calcium imaging and controls for loose-patch recordings with ChR2 activation.** (A) Illustration of the experimental system: spontaneous two-photon calcium imaging recordings from an awake, head-fixed mouse on a treadmill (see S1 Text). (B) The expression of ChR2 in a VChI and GCaMP6s in the local cortical circuitry. (C) Example of a non-VChI ROI response (green cell) to VChI stimulation. Following activation, we observed a clear “rebound” response in cells affected by VChI activation (similar rebound was also observed in Lee et al. [87]; arrow: light stimulation). (D) Comparison of the average events per second for all ROIs included in analysis (*n =* 280; red cells are excluded). The distribution indicates an overall rebound effect following light stimulation (a random jitter at the order of 0.005 was added to each point for visualization). (E) Illustration of the experimental system: spontaneous cell-attached recordings from an awake, head-fixed mouse on a treadmill. (F) Spiking activity of a cell in response to VChI stimulation. During light activation, the cell was inhibited; this was followed by a rebound response when the light was switched off (100 ms-bin; dashed red line: mean baseline activity). (G) Population PSTH (*n =* 29; 250-ms bin). As in panel F, rebound followed inhibition. (H) Average whisking 1 s before and during masking light activation (pre mask: 0.45 ± 0.03; during mask: 0.46 ± 0.03; *n =* 8 mice; paired *t* test, *t*[7] = 2.16, *p =* 0.07). (I) Rate change (spikes per second) of cells during masking light activation (0.2 ± 0.3 spikes/s). ROI, region of interest.
(TIF)

S1 Text Materials and methods related to S2A–S2D Fig. (DOCX)

S1 Data Data used for summary plots in all figs. (XLSX)

## Abbreviations

τ_m_: membrane time constant
ACh: acetylcholine
BF: basal forebrain
ChR2: channelrhodopsin2
ChAT: choline acetyltransferase
CI: convergence index
GABA: γ-aminobutyric acid
GFP: green fluorescent protein
HW: high whisking
IACUC: Institutional Animal Care and Use Committee
IPSC: inhibitory postsynaptic current
LFP: local field potential
LW: low whisking
MSN: medium spiny neuron
NDS: Normal Donkey Serum
oG: optimized glycoprotein
PCR: polymerase chain reaction
PSTH: peristimulus time histogram
PW: principal whisker
Pyr: pyramidal neuron
R_in_: input resistance
RNA-seq: RNA sequencing
S2: secondary somatosensory cortex
SST: somatostatin
TVA: avian tumor virus receptor A
V_th_: threshold potential
VChI: VIP/ChAT interneuron
VIP: vasoactive intestinal polypeptide
VPM: ventral posteromedial thalamic nucleus
WT: wild-type
