# Supplementary figures and images for: Barrel cortex VIP/ChAT interneurons suppress sensory responses in vivo
Source: PLoS Biol. 2020 Feb 6;18(2):e3000613. doi: 10.1371/journal.pbio.3000613 (PMC7029879; doi:10.1371/journal.pbio.3000613)

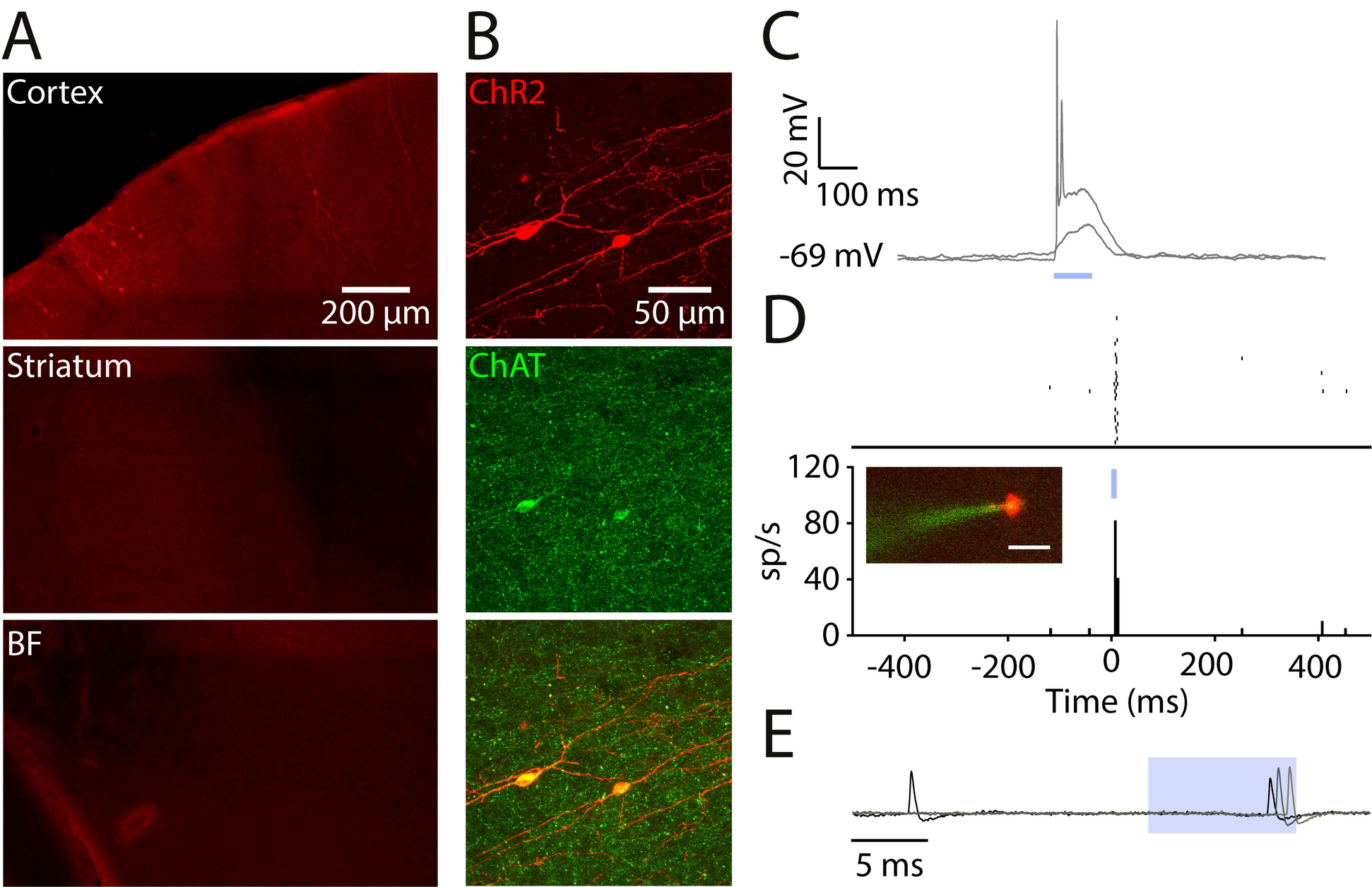

Supplement: S1 Fig — VChIs reliably express ChR2. (A) Expression of a Cre-dependent ChR2-tdTomato virus in a ChAT-Cre mouse following injection to the barrel cortex (areas: cortex, striatum, and BF). (B) Immunostaining for ChAT (Alexa 647) in a ChAT-Cre mouse expressing ChR2-tdTomato. (C) In vitro responses to light stimulation of a ChR2-expressing neuron. An intensity of 1 mW/mm2 evoked a spiking response, whereas 0.1 mW/mm2 yielded only subthreshold depolarization. (D) In vivo targeted cell-attached recording from a VChI expressing ChR2. The raster plot (top) and PSTH (bottom; 5-ms bin) show sharp elevation of firing rate in response to light stimulation. Mean-intensity z-projection of the targeted cell (scale: 20 μm). (E) Three trials from panel D (blue area: light stimulation). (TIF) [file pbio.3000613.s002.tif]

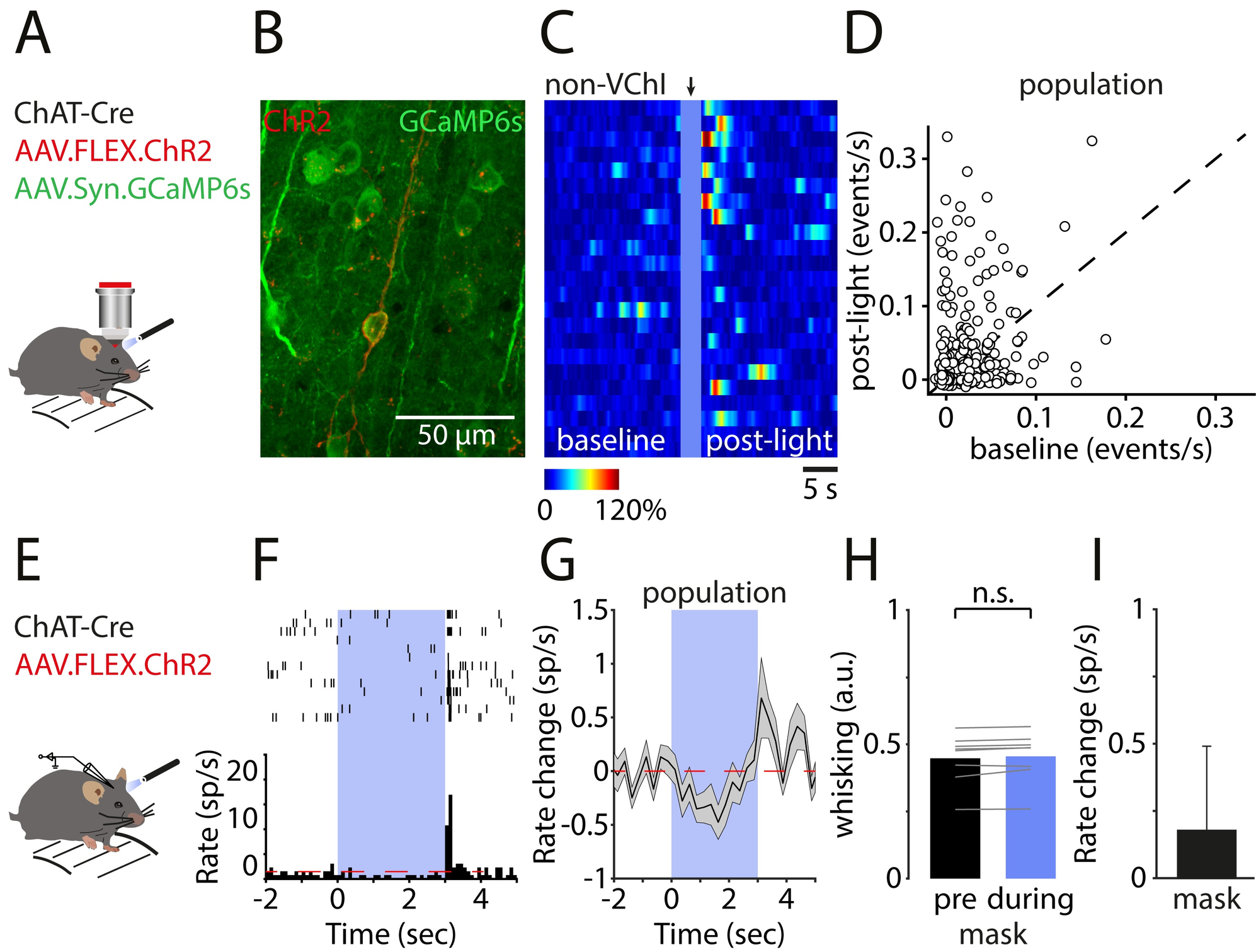

Supplement: S2 Fig — Two-photon calcium imaging and controls for loose-patch recordings with ChR2 activation. (A) Illustration of the experimental system: spontaneous two-photon calcium imaging recordings from an awake, head-fixed mouse on a treadmill (see S1 Text). (B) The expression of ChR2 in a VChI and GCaMP6s in the local cortical circuitry. (C) Example of a non-VChI ROI response (green cell) to VChI stimulation. Following activation, we observed a clear “rebound” response in cells affected by VChI activation (similar rebound was also observed in Lee et al. [87]; arrow: light stimulation). (D) Comparison of the average events per second for all ROIs included in analysis (n = 280; red cells are excluded). The distribution indicates an overall rebound effect following light stimulation (a random jitter at the order of 0.005 was added to each point for visualization). (E) Illustration of the experimental system: spontaneous cell-attached recordings from an awake, head-fixed mouse on a treadmill. (F) Spiking activity of a cell in response to VChI stimulation. During light activation, the cell was inhibited; this was followed by a rebound response when the light was switched off (100 ms-bin; dashed red line: mean baseline activity). (G) Population PSTH (n = 29; 250-ms bin). As in panel F, rebound followed inhibition. (H) Average whisking 1 s before and during masking light activation (pre mask: 0.45 ± 0.03; during mask: 0.46 ± 0.03; n = 8 mice; paired t test, t[7] = 2.16, p = 0.07). (I) Rate change (spikes per second) of cells during masking light activation (0.2 ± 0.3 spikes/s). ROI, region of interest. (TIF) [file pbio.3000613.s003.tif]
