# Supplementary material for: Barrel cortex VIP/ChAT interneurons suppress sensory responses in vivo
Source: PLoS Biol. 2020 Feb 6;18(2):e3000613. doi: 10.1371/journal.pbio.3000613 (PMC7029879; doi:10.1371/journal.pbio.3000613)
Supplement: S1 Text — (DOCX) [file pbio.3000613.s004.docx]

**S1 Text.** Materials and methods related to S2A-D Fig. Viral injection was done in the same method as described in the main Materials and methods section. A 500 nl mix of a AAV1-CAGGS-Flex-rev-ChR2-tdTomato (1 × 10^13^ genomic copies per mL; Atasoy et al., 2008) and a pAAV1-Syn-GCaMP6s (3 × 10^13^ genomic copies per mL; Chen et al., 2013) was injected into the barrel cortex of a ChAT-Cre mouse. 3 wk post-injection, we installed a headpost and made a 3-mm craniotomy above the injection site. Then, we placed an imaging window for covering the brain surface. Mice were head-fixed on a treadmill 1–2 h before the imaging session. Imaging was performed with a low-power temporal oversampling (LOTOS) two-photon microscope (Suzhou Institute of Biomedical Engineering and Technology) at 920 nm with a Ti:Sapphire laser (Vision II, Coherent, Santa Clara, CA) and imaged through a 16X, 0.8 NA water immersion objective (Nikon, Tokyo, Japan). Full-frame images (600 × 600 pixels; 40 Hz frame rate) were taken from layer 2/3 expressing GCaMP6s at depths of 150–200 μm below the cortical surface. The optogenetic activation was performed through optic fiber approximately 2 mm above the cortical surface with 473 nm laser (diameter 200 μm, 0.39 NA; 15 mW/mm^2^; Thorlabs, Newton, NJ). Every 60 s we activated the VChIs with a 3-s continuous stimulation. During this activation time, the two-photon photomultipliers (PMTs) received near-zero voltage for functionally deactivating them. Then, 500 ms following the end of the stimulation, we reactivated the PMTs. All data were acquired using custom-built software based on LabVIEW (National Instruments, Austin, TX).

Analyses were performed using ImageJ software (Schneider et al., 2012) and MATLAB (MathWorks, Natick, MA). Only trials in which mice running velocity was < 1 cm/s either 30 s before or after light stimulation were picked up for analysis. We corrected for x-y movements using the “moco” plugin (Dubbs et al., 2016), and manually selected regions of interest (ROIs) for the neurons. ROIs that expressed both GCaMP6s and ChR2 (tdTomato-expression) were omitted from the analysis. For event detection analysis, we focused on large fluorescent changes. We smoothed the fluorescence traces with a 0.25 s sliding window, and the percent change in fluorescence signal was calculated using (F − F_0_) / F_0_. The F_0_ for each ROI was defined by the mode of the fluorescence histogram of the ROI; however, in highly active ROIs, F_0_ was defined as the fifth percentile of the ROI’s fluorescence histogram. ROIs with (F − F_0_) / F_0_ < 0.25 throughout all the experiment were omitted from analysis. Event was defined as a rise above 50% prominence of the (F − F_0_)/F_0_ signal. For each cell, the average number of events across all trials was calculated in a 2 s window pre- and post-light stimulation.

Atasoy D, Aponte Y, Su HH, Sternson SM. A FLEX switch targets channelrhodopsin-2 to multiple cell types for imaging and long-range circuit mapping. J Neurosci. 2008;28: 7025–7030. doi:10.1523/JNEUROSCI.1954-08.2008

Chen TW, Wardill TJ, Sun Y, Pulver SR, Renninger SL, Baohan A, et al. Ultrasensitive fluorescent proteins for imaging neuronal activity. Nature. 2013;499: 295-300. doi:10.1038/nature12354

Schneider CA, Rasband WS, Eliceiri KW. NIH Image to ImageJ: 25 years of image analysis. Nat Methods. 2012;9: 671–675. doi:10.1038/nmeth.2089

Dubbs A, Guevara J, Yuste R. moco: Fast motion correction for calcium imaging. Front Neuroinform. 2016;10: 6. doi:10.3389/fninf.2016.00006
